# The effect of renal denervation in an experimental model of chronic renal insufficiency, The REmnant kidney Denervation In Pigs study (REDIP study)

**DOI:** 10.1186/s12967-017-1319-0

**Published:** 2017-10-25

**Authors:** Jean-Claude Lubanda, Miroslav Chochola, Mikuláš Mlček, Petr Neužil, Josef Marek, Štěpán Havránek, Sylvie Kuchynková, Zdeňka Fingrová, Kao-Hsuan Aimee Huang, Aleš Linhart

**Affiliations:** 1 2nd Department of Medicine-Department of Cardiovascular Medicine, First Faculty of Medicine, Charles University in Prague and General University Hospital in Prague, U Nemocnice 2, 128 00 Prague 2, Czech Republic; 2 Institute of Physiology, First Faculty of Medicine, Charles University in Prague, Albertov 5, 128 00 Prague 2, Czech Republic; 3 Department of Cardiology, Na Homolce Hospital, Roentgenova 2/37, 150 30 Prague 5, Czech Republic

**Keywords:** Renal sympathetic denervation, Renal insufficiency, Renal ischemia, Remnant kidney porcine model, Renal artery embolization, Chronic kidney disease

## Abstract

**Background:**

Renal denervation (RDN) is a promising therapeutic method in cardiology. Its currently most investigated indication is resistant hypertension. Other potential indications are atrial fibrillation, type 2 diabetes mellitus and chronic renal insufficiency among others. Previous trials showed conflicting but promising results, but the real benefits of RDN are still under investigation. Patients with renal insufficiency and resistant hypertension are proposed to be a good target for this therapy due to excessive activation of renal sympathetic drive. However, only limited number of studies showed benefits for these patients. We hypothesize that in our experimental model of chronic kidney disease (CKD) due to ischemia with increased activity of the renin–angiotensin–aldosterone system (RAAS), renal denervation can have protective effects by slowing or blocking the progression of renal injury.

**Methods:**

An experimental biomodel of chronic renal insufficiency induced by ischemia was developed using selective renal artery embolization (remnant kidney porcine model). 27 biomodels were assessed. Renal denervation was performed in 19 biomodels (denervated group), and the remaining were used as controls (n = 8). The extent of renal injury and reparative process between the two groups were compared and assessed using biochemical parameters and histological findings.

**Results:**

Viable remnant kidney biomodels were achieved and maintained in 27 swine. There were no significant differences in biochemical parameters between the two groups at baseline. Histological assessment proved successful RDN procedure in all biomodels in the denervated group. Over the 7-week period, there were significant increases in serum urea, creatinine, and aldosterone concentration in both groups. The difference in urea and creatinine levels were not statistically significant between the two groups. However, the level of aldosterone in the denervated was significantly lower in comparison to the controls. Histological assessment of renal arteries showed that RDN tends to produce more damage to the arterial wall in comparison to vessels in subjects that only underwent RAE. In addition, the morphological damage of kidneys, which was expressed as a ratio of damaged surface (or scar) to the overall surface of kidney, also did not show significant difference between groups.

**Conclusions:**

In this study, we were not able to show significant protective effect of RDN alone on ischemic renal parenchymal damage by either laboratory or histological assessments. However, the change in aldosterone level shows some effect of renal denervation on the RAAS system. We hypothesize that a combined blockade of the RAAS and the sympathetic system could provide more protective effects against acute ischemia. This has to be further investigated in future studies.

## Background

Renal denervation (RDN) is a promising therapeutic method in cardiology [1]. Potential indications for renal denervation are treatments for resistant hypertension, sleep apnea syndrome, insulin resistance, atrial fibrillation, ventricular tachycardia, and chronic renal insufficiency [2, 3]. Initially, Symplicity HTN-1, Symplicity HTN-2, and other smaller trials showed promising results of RDN as a potential treatment modality [4–7]. However, Symplicity HTN-3 trial showed less encouraging results; it confirmed the safety of the technique without proving the efficacy of renal denervation as a treatment option [8]. Nonetheless, the real benefits of this method are still under investigations. Recent studies showed moderate to equivocal effects in specific study populations [9, 10]. Patients with renal insufficiency and resistant hypertension were proposed to be the best target population for this therapy due to excessive activation of renal sympathetic drive [11]. Very few reports exist that show benefits for these patients. In our experimental model of Chronic Kidney Diseases (CKD) established by induced ischemia, we hypothesize that renal sympathetic denervation can have protective effects by slowing or blocking the progression of renal injury. The aim of our study was to assess the effects of RDN on the progression of chronic renal insufficiency through an experimental model of CKD. The extent of renal injury and reparative process were assessed using laboratory parameters and histology.

## Methods

We used the porcine remnant kidney model of chronic renal insufficiency that was developed by Misra and his coworkers [12]. This biomodel was created using renal artery embolization with PVA particles. A stable and reproducible model of renal insufficiency in swine developed by the 4th week and lasted up to 12 weeks. Total left and partial right nephrectomies were performed to develop this model. Acute deterioration of renal functions ensued after embolization, but improves and stabilizes soon after. Renal insufficiency was later confirmed by a statistically significant increase in creatinine, BUN, and transient changes in blood pressure. The remnant kidney eventually developed fibrosis in the tubulointerstitial compartment as it hypertrophies in the late stage.

Cross-bred swine (Landrace × White; 44 ± 3 kg) were used for this experiment in an accredited university laboratory by a skilled team of clinicians and veterinary specialists. The animals were handled in accordance with the guidelines of research animal use [13]. The protocol was approved by the Charles University 1st Faculty of Medicine Institutional Animal Care and Use Committee, and the procedures were performed at the Animal Laboratory, Institute of Physiology, 1st Faculty of Medicine, Charles University in Prague in accordance with Act No 246/1992 as amended, Collection of Laws, Czech Republic, and EU Directives 86/609/EEC as amended, 2007/526/ES, 2010/63/EU.

The experiment was conducted on a total of 27 swine. The population was divided into two groups: a control group (n = 8) and a denervated group (n = 19). Experimental animals underwent general anesthesia, and after renal and hemodynamic parameters had been collected to establish baseline parameters, 19 animals (denervated group) underwent renal denervation in addition to renal artery embolization (RAE) done on all 27 animal subjects. After the procedure, data on renal function, hemodynamics, serum renin and aldosterone were obtained. After 42 days, post-procedural follow-ups and re-tests of renal and hemodynamic parameters were conducted, the swine were then euthanized for histological assessments. One to two procedures were performed monthly; all procedures on swine were concluded during the first 24 months of study, then data analyses were performed the year after.

### Anesthesia and monitoring

The pigs were sedated by intramuscular application of azaperone (2–3 mg/kg) and ketamine (20 mg/kg). The marginal ear vein was cannulated, and general anesthesia was induced by an intravenous bolus of propofol (1–2 mg/kg). After preoxygenation via facial mask, orotracheal intubation was performed. During the experiment, biomodels were mechanically ventilated using Intellivent-ASV closed-loop system (G5, Hamilton Medical, Bondauz, Switzerland) to maintain normoxia (SpO_2_ 98%) and normocapnia (EtCO_2_ 38–40 mmHg) respective to the actual metabolic rate. The total intravenous anesthesia was maintained by continuous administration of propofol (6–12 mg/kg/h) and morphine (0.1–0.2 mg/kg/h). The depth of anesthesia was regularly assessed by photoreaction and corneal reflex and adjusted accordingly. Intravenous infusion of Ringer’s solution was given to reach and maintain central venous pressure between 6 and 8 mmHg. Anticoagulation was provided by unfractionated heparin bolus (100 IU/kg IV), followed by continuous intravenous drip (40–50 IU/kg/h) to maintain target activated clotting time of 180–250 s (values checked every hour with Hemochron Junior+, International Technidyne Corporation, Edison, NJ, USA). Sheaths and catheters were inserted into femoral and carotid/jugular vessels as needed. Invasive blood pressure from carotid and pulmonary arteries, central venous pressure (TruWave, Edwards Lifesciences, USA), body surface ECG, capnometry and pulse oximetry were continuously monitored by bedside monitor (Life Scope TR, Nihon Kohden, Japan). All procedures were performed under completely sterile environment to avoid infection and contamination. This technique used for anesthesia and monitoring has been described in our previous publication [14].

### Renal denervation

After initiation of general anesthesia and mechanical ventilation, an 8 French sheath was placed into the right common femoral artery under ultrasound guidance, and 6 French sheath was then introduced into the right common femoral vein. The later was used for the acquisition of samples for biochemical analysis during the procedure. According to the protocol, after preliminary samples were withdrawn, renal angiography was performed, and an ablation catheter was then introduced into the renal arteries. In 6 cases, the EnligHTN™ catheter (St. Jude Medical, USA) was used, in 8 cases we used the Symplicity catheter (Medtronic, USA), and in 5 cases the ThermoCool^®^ catheter (Biosense Webster) was used. The ablation procedure was performed according to the instructions for the use of each device in both arteries in each case, and the effect of the procedure was controlled by a drop of impedance of at least 10% (Table 1). Finally, the femoral sheath was removed and the femoral artery was surgically sutured. After this, the administration of anesthetics and analgesics ceased and successful weaning from artificial ventilation was achieved. The animals were then extubated. For the next 42 days, the animals were bred in a certified menagerie.

**Table 1:** Denervation characteristics

Group	Median [25th–75th percentile]	Mean ± SD
Watt max	8.0 [6.0–10.7]	8.8 ± 3.6
Watty average	7.2 [4.8–9.9]	7.6 ± 3.9
Temperature max	70 [55–75]	65 ± 12
Temperature average	60 [54–68]	59 ± 11
Time max	120 [90–120]	110 ± 15
Time average	117 [90–120]	108 ± 14
Impedance change right	0.118 [0.072–0.159]	0.119 ± 0.052
Impedance change left	0.147 [0.090–0.165]	0.133 ± 0.049
Impedance change average	0.127 [0.097–0.153]	0.127 ± 0.045
Type of device		
ThermoCool^®^	5 (26%)	
EnligHTN™	6 (32%)	
Symplicity	8 (42%)	

### Renal artery embolization

Selective angiography of the renal artery was performed using a predefined protocol. Using a guiding catheter, the left renal artery was cannulated and 40 ml of Bead Block^®^ (BTG Inc.) (300–500 μm) was applied; the left renal artery had to be completely occluded. Subsequently, the right renal artery was also cannulated, and the right upper branch was used as the target vessel where 20 ml of Bead Block (300–500 μm) was injected to achieve partial nephrectomy. Control angiography was performed showing complete occlusion of the left renal artery and partial occlusion of the right upper branch.

### Statistics

The sample size was calculated using Medcalc software (Medcalc^®^ Version 12,1.4.0) based on the primary outcome of the study, which was the difference of creatinine concentration between the denervated and the control group at follow-up. The minimal required sample size per group for equal samples sizes for alpha = 0.05 and power = 0.80 (beta = 0.20) was 15 per group, assuming that the data will be normally distributed.

Continuous data are presented as mean ± SD or median [25th–75th percentile] as appropriate. Categorical data are presented as proportions. Due to the non-normal distribution of several continuous variables, nonparametric Mann–Whitney U test was used to compare baseline variables and histology data. Baseline categorical variables were compared using the Chi square test. Change of laboratory values in time, comparisons between groups at follow-up and differences in change of laboratory values was assessed using linear mixed modeling; serum creatinine and blood urea values were log-transformed for the analysis to better accommodate the linear model assumptions. p < 0.05 were considered significant. Statistical analysis and plotting were performed using R software, version 3.2.3 (R Foundation for Statistical Computing, Vienna, Austria).

## Results

### RDN procedure parameter

Main parameters of renal denervation are shown in Table 1. The mean energy delivered to the tissues were sufficient, and the increase in temperature was adequate. The drop in impedance showed that all radiofrequency ablation with all three devices were effective.

### Biochemical changes

The baseline characteristics of the study population are recapitulated in Table 2. There was no significant difference in renal parameters between groups (denervated vs controls: Urea 3.10 [2.85–3.27] mmol/l vs. 3.10 [2.47–3.80] mmol/l, p = NS, creatinine 109 [104–128] µmol/l vs. 118 [93–126] µmol/l, p = NS). We were able to develop the ischemic model in all surviving animals.

**Table 2:** Baseline data

Group	Denervation		Control		p
	Median [25th–75th percentile]	Mean ± SD	Median [25th–75th percentile]	Mean ± SD	
Finished experiment	13 (68%)		5 (62%)		0.77
Baseline biochemistry					
Sodium (mmol/l)	138.0 [133.5–138.8]	135.8 ± 4.3	141.0* [138.0–143.0]	141.3 ± 4.0*	0.02
Potassium (mmol/l)	3.60 [3.50–3.85]	3.70 ± 0.25	3.30 [3.10–3.60]	3.38 ± 0.36	0.05
Chloride (mmol/l)	97.0 [94.0–98.5]	96.5 ± 3.4	102.0* [99.2–104.0]	101.7 ± 3.6*	0.004
Urea (mmol/l)	3.10 [2.85–3.27]	3.05 ± 0.31	3.10 [2.47–3.80]	3.21 ± 1.11	0.77
Creatinine (μmol/l)	109 [104–128]	118 ± 25	118 [93–126]	113 ± 20	0.80
Renin (pg/ml)	0.30 [0.30–0.30]	0.25 ± 0.10	0.30 [0.20–0.70]	0.53 ± 0.59	0.79
Aldosteron (ng/l)	5.0 [5.0–5.0]	4.3 ± 2.1	5.0 [4.2–5.5]	5.9 ± 5.0	0.83

* p < 0.05 vs. denervation

The creatinine level estimate increased from baseline to maximal value, and finally remained at a high level in both groups as shown in Table 4. Creatinine values at 7th week were significantly higher both compared to Baseline and 4th week. This shows that we were able to establish a successful model of renal ischemia through renal artery embolization, and we managed to sustain the viability of a large group of animal subjects over a long period of time (Table 3). Although serum sodium and chloride levels were observed to be different among the two groups; overall, the baseline p values of most serum compounds in controls and denervated show that there were no significant differences among the Denervated group and the Controls at baseline (Table 2).

**Table 3:** Survival rate

	Baseline	Early termination	Completed experiment
Denervation	19	6	13 (68%)
Control	8	3	5 (62%)
Total	27	9	18 (66%)

**Fig. 1:**
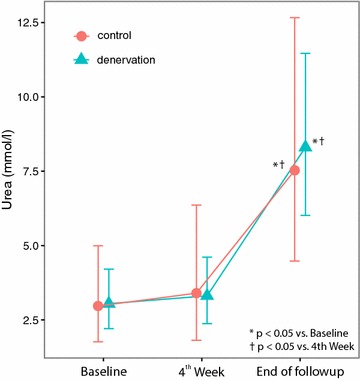
Comparison of serum level of urea between groups (baseline/4th week/follow up)

**Fig. 2:**
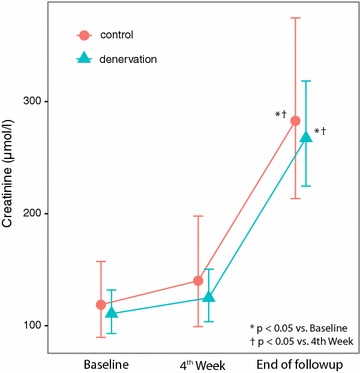
Comparison of serum level of creatinin between groups (baseline/4th week/follow up)

**Fig. 3:**
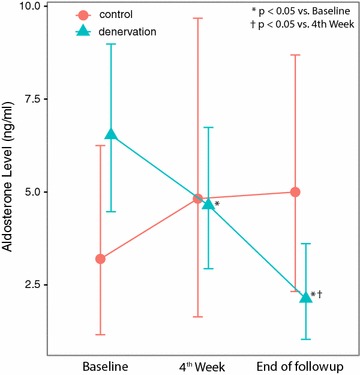
Comparison of serum aldosterone level between groups (baseline/4th week/follow up)

Over the 7-week period, there were significant changes in serum urea, creatinine, and aldosterone concentration in both groups, but the difference between two groups was not statistically significant for urea and creatinine levels at both week 4 and week 7 follow-up. Furthermore, there was no significant difference in change of the values over time (Table 4, Figs. 1, 2). This shows that renal denervation did not provide specific protection to prevent renal parenchymal damage by ischemia. However, the level of aldosterone in the denervation group decreased from baseline and was significantly lower in comparison to the control group. This shows that renal denervation could have a mild protective effect by interfering with the renin–angiotensin–aldosterone (RAAS) system (Fig. 3).

**Table 4:** Laboratory Values at Follow up, n = 18 (denervated n = 13, control n = 5)

Variable	Group	Baseline Coef [95% CI]	Week 4 Coef [95% CI]	End of study Coef [95% CI]
Creatinine (μmol/l)	Control	119 [90–157]	140 [99–198]	283*^†^ [214–375]
	Denervation	111 [93–132]	125 [104–150]	267*^†^ [225–318]
Urea (mmol/l)	Control	2.97 [1.77–4.99]	3.40 [1.82–6.36]	7.53*^†^ [4.48–12.67]
	Denervation	3.05 [2.21–4.21]	3.31 [2.38–4.61]	8.30*^†^ [6.02–11.47]
Aldosteron (ng/l)	Control	3.20 [1.16–6.25]	4.82 [1.64–9.68]	5.00 [2.32–8.68]
	Denervation	6.53 [4.47–8.98]	4.64 [2.94–6.74]	2.13*^†^ [1.03–3.61]

Estimates are based on linear mixed models

*Coef* coefficient

* p < 0.05 vs. baseline

^†^p < 0.05 vs. 4th week. No significant differences between groups were observed at any time point

### Histology findings

The histological findings in renal arteries are shown in Table 5. We assessed the level of injuries in each layer of the arterial wall according to the histologic vascular injury grading scale (0–5) [15]. As seen in the table, arteries without renal denervation tend to have less damage compared to the denervation group since no manipulation to the artery was applied apart from renal artery embolization.

**Table 5:** Histology data

Group	Control group median [25th–75th percentile]	Denervated group median [25th–75th percentile]	p
Arterial wall (histologic vascular injury grading scale, 0–5)			
Intima	1.50 [1.00–1.50]	3.00* [2.00–3.75]	0.0337
Lamina elastica interna	1.00 [1.00–1.00]	1.50* [1.00–2.12]	0.0464
Media	0.0 [0.0–0.5]	2.5* [2.0–3.0]	0.0106
Lamina elastica externa	0.00 [0.00–0.50]	2.50* [2.00–2.62]	0.0047
Adventitia	0.0 [0.0–0.5]	2.5* [2.4–2.6]	0.0029
Kidney scarring (ratio of damaged surface (or scar) to overall surface of the kidney (%)	25 [24–87]	45 [35–62]	0.4228

* p < 0.05 vs. control group

**Fig. 4:**
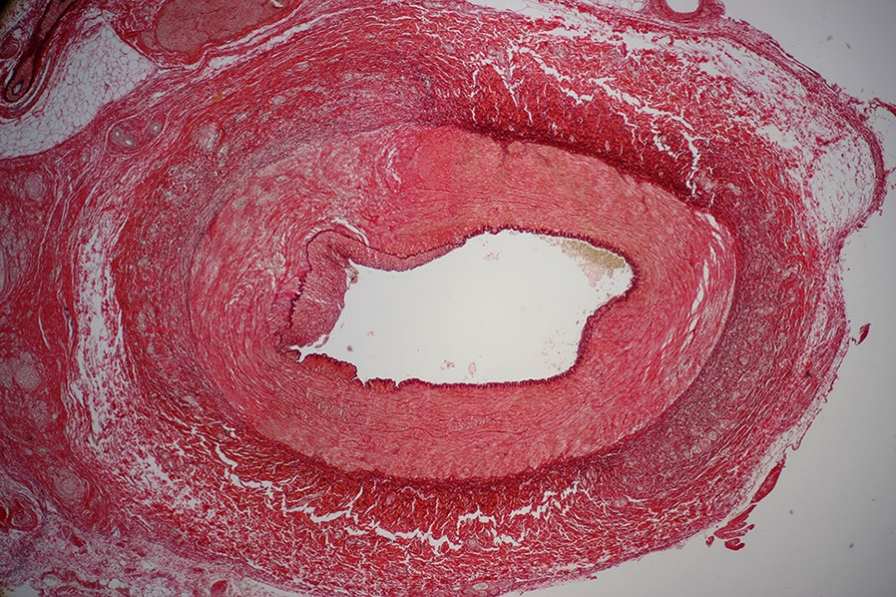
A cross-section of the denervated renal artery showing increased inflammation around the intima and destroyed nerves

**Fig. 5:**
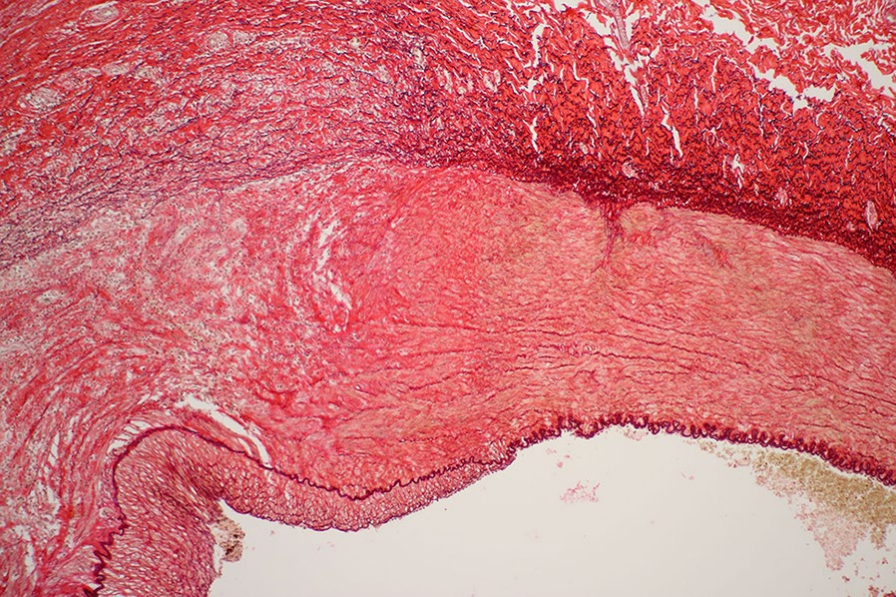
Detail of a cross-section of the denervated renal artery showing increased inflammation around the intima and destroyed nerves

A cross-section of the denervated renal artery can be seen with increased inflammation around the intima and the destroyed nerves (Figs. 4, 5). The morphological damage of kidneys expressed as a ratio of damaged surface (or scar) to overall surface of the kidney also did not show significant difference between groups (Table 5).

## Discussion

Renal denervation is a therapeutic method which was expected to impact the treatment of chronic renal insufficiency [16, 17]. This concept is widely accepted because the increase of sympathetic activity is one of the important factors that contributes to chronic kidney damage [18, 19]. Experimental studies with stimulation of the sympathetic system have shown increase release of renin–angiotensin–aldosterone and other agents from kidney. Moreover, markers of sympathetic activity such as MSNA, noradrenaline, and neuropeptide Y, are elevated in CKD patients on average, but show considerable variation in different studies [20, 21]. Thus, we hypothesize that blocking sympathetic system by RDN would have a protective effect against renal damage caused by ischemia. First, we developed a sustained biomodel of chronic renal insufficiency on which we could then test our hypothesis. Developing a sustained biomodel was a considerable achievement considering the risk of loss-of-animal during the acute phase. The increase in serum creatinine was dramatic after the initial renal embolization. However, there were no significant differences in changes of urea and creatinine levels between the controls and the denervated group; this may be because the initial ischemia causes dramatic increases in the sympathetic activity during the acute phase, and renal denervation has only a limited effect on that massive hyperactivity. RDN alone is not sufficient to curb that hyperactivation [17, 22, 23]. However, a minor change was observed in the denervated group (the lower of aldosterone level), which shows that renal denervation could have a mild protective effect through its interference with the renin–aldosterone–angiotensin (RAAS) system. This has to be investigated further in future studies. It will be of interest to investigate the combined effect of RAAS blockade and RDN on this biomodel. Moreover, newer techniques used to modulate the sympathetic system are emerging, such as carotid body ablation or barostimulation [24–29]. The effects of these techniques as potential treatment modalities for resistant hypertension and chronic renal insufficiency also need to be investigated further.

## Study limitation

In our study, we were not able to measure renin level at follow up because of technical problems therefore we cannot reliably comment on that parameter although we hypothesized that renin levels would have shown the same trend as aldosterone. Also, the number of controls which was planned to be higher was subsequently limited because of our preliminary results showing consistent trend in both groups. After interim analysis, it was clear that adding more controls will not increase the statistical power of the experiment. Another limitation of our study is that Renal denervation and renal artery embolization were performed during baseline at the same time point which made controlling excessive activation of the sympathetic system by acute ischemia impossible. Interestingly, it would be quite challenging to limit the effects of acute ischemia by establishing the CKD biomodel first and then perform RDN when the renal insufficiency is stable. This would require also a longer duration of the whole experiment. This approach would eliminate the acute effects of ischemia and uncover the real effects of renal denervation on chronically increased sympathetic drive which is not the case in the present study.

## Conclusions

With this experiment, we were able to establish an experimental model of chronic renal insufficiency by embolization of renal artery. Unfortunately, we were not able to show significant protective effects of renal denervation alone on renal parenchyma damage caused by ischemia. However, the change in aldosterone level shows some effect of renal denervation on the RAAS system. We hypothesize that a combined blockade of the RAAS and the sympathetic system might be more protective against acute ischemia, but this has to be investigated further in future studies.

## Abbreviations

RAAS: renin–angiotensin–aldosterone system
CKD: chronic kidney diseases
RDN: renal sympathetic denervation
RAE: renal artery embolization
